# A case report of pulmonary atresia with intact ventricular septum: an extraordinary finding of subsystemic right ventricle

**DOI:** 10.3389/fped.2024.1251274

**Published:** 2024-05-01

**Authors:** Gang Luo, Ai Liu, Hongxiao Sun, Kuiliang Wang, Silin Pan

**Affiliations:** Heart Center, Women and Children’s Hospital, Qingdao University, Qingdao, China

**Keywords:** pulmonary atresia with intact ventricular septum, tricuspid regurgitation, echocardiography, fetus, case report

## Abstract

**Background:**

Massive tricuspid regurgitation (TR) is the most common feature of pulmonary atresia with intact ventricular septum (PA/IVS), and mild or absent TR is observed in severe right ventricular (RV) dysplasia or RV-to-coronary fistulous connections, resulting in non-biventricular (BV) outcomes postnatally.

**Case summary:**

We report a case of fetal severe pulmonary stenosis with IVS diagnosed at 26 weeks of gestation. The severity of RV hypoplasia did not worsen or reach indications for intrauterine intervention, while the jet velocity of TR decreased significantly during pregnancy. The fetus was definitely diagnosed with PA/IVS with mild RV dysplasia after birth. Unusually, the fetus did not experience severe TR and myocardial sinusoids, the TR jet velocity was maintained at 2.0 m/s, and the coronary artery was almost normal. The incapable RV cannot pump blood into pulmonary circulation after RV decompression from valvular perforation and balloon dilation. It may be an extraordinary finding of subsystemic RV.

**Conclusion:**

PA/IVS is a heterogeneous disease with various degrees of RV dysplasia. Mild or no baseline TR is a reliable indicator with non-BV outcomes for fetal PA/IVS, even with acceptable dysplasia RV structures.

## 1 Introduction

Severe pulmonary stenosis or atresia with intact ventricular septum (PA/IVS) is a rare form of complex congenital heart disease characterized by hypoplasia of the right ventricle (RV) and the tricuspid valve (TV). As a heterogeneous disease, there are various associated factors with postoperative results in PA/IVS, including RV size and morphology, TV size and function, and the right ventricle-dependent coronary circulation (RVDCC). It remains challenging to determine the appropriate surgical approach for patients with moderate developmental indicators, unlike those with severe or mild dysplasia. There is no exact regulation for the development of pathological modification in fetal PA/IVS. Currently, the evaluation of RV function in fetal PA/IVS remains challenging. With the conviction that good structure can result in improved function, structural markers, such as the TV size and the ratio of the left and right ventricles on fetal echocardiography, may provide useful intrauterine indications to the postpartum outcome of PA/IVS (1).

Tricuspid regurgitation (TR) can be readily identified during a regular obstetric ultrasound, making it a good indicator of RV function during pregnancy. A multicenter study indicates that fetuses with mild or no baseline TR may not be able to achieve biventricular (BV) outcomes due to the presence of severe RV dysplasia or RVDCC (2). Only by opening the pulmonary valve can the “true capacity” of RV to pump blood to the pulmonary artery be truly revealed, though the results may not always meet expectations, as found in clinical research (3). We observed a fetus diagnosed with severe pulmonary stenosis with IVS at 26 weeks of gestation whose TR jet velocity decreased during gestation in a manner that was not expected based on the cardiac structural assessment, an extraordinary type of deterioration in RV function, and a postnatal outcome that did not achieve BV repair.

## 2 Methods and results

### 2.1 Case presentation

**Figure 1:**
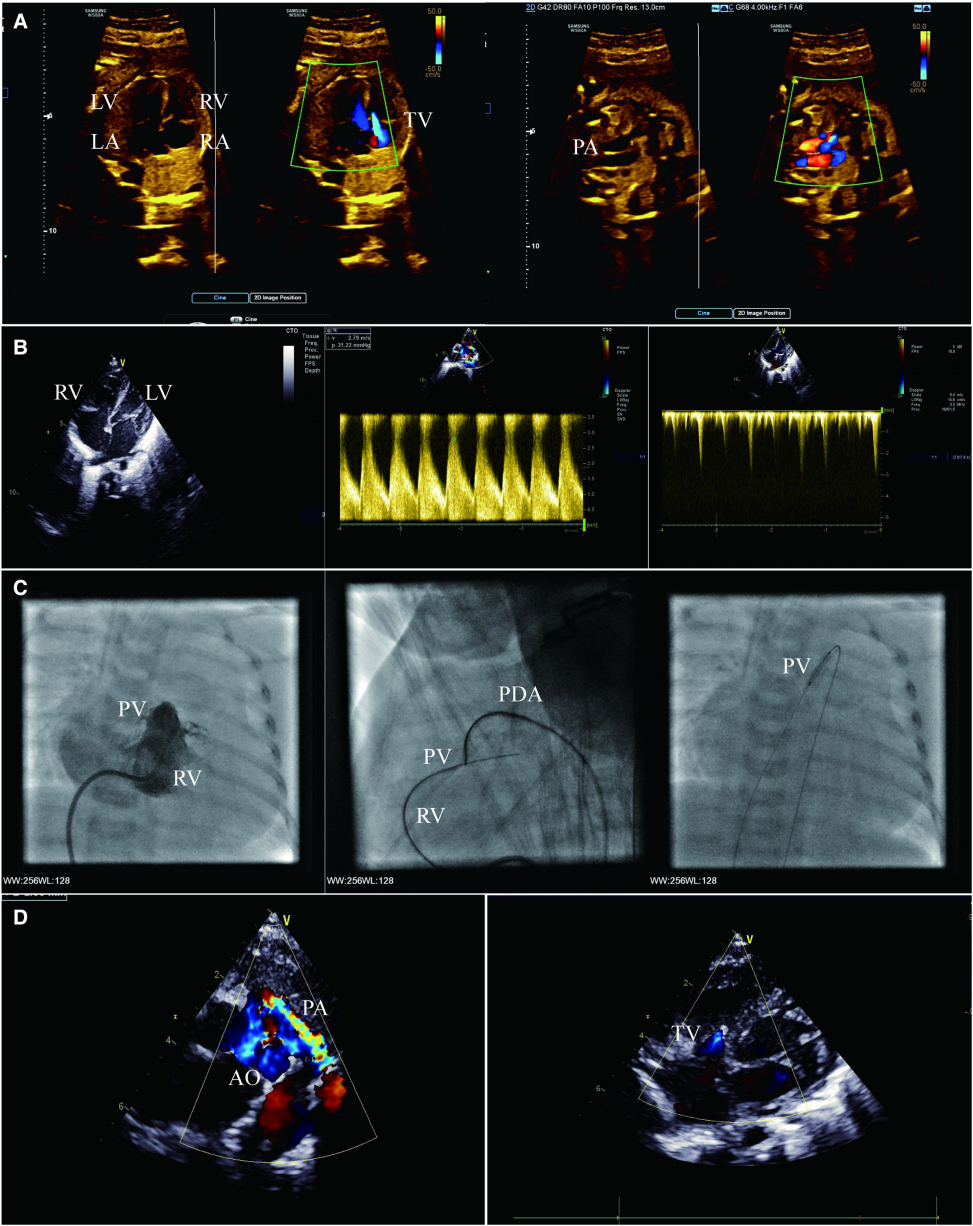
(**A**) Fetal echocardiogram apical four-chamber view (left) and right ventricular outflow tract view (right) showing severe pulmonary stenosis and massive tricuspid regurgitation. (**B**) Transthoracic echocardiogram apical four-chamber view (left and right) and short-axis view (middle) showing mild hypoplasia of RV, pulmonary atresia and patent ductus arteriosus, no serious abnormality of TV, and mild tricuspid regurgitation. (**C**) Right ventriculography (left) showing mild hypoplasia of RV and pulmonary valvular atresia. Lateral right ventriculography (middle) showing guide wire creating an arteriovenous wire loop. Anterior chest fluoroscopy (right) showing percutaneous balloon pulmonary valvuloplasty. (**D**) Transthoracic echocardiogram short-axis view (left) and apical four-chamber view (right) showing ductus arteriosus supply pulmonary blood flow, no antegrade blood flow across the pulmonary valve, and mild tricuspid regurgitation. LA, left atrium; LV, left ventricle; PV, pulmonary valve.

A 36-year-old woman underwent a routine prenatal examination at 26 weeks of pregnancy at the Women and Children's Hospital, Qingdao University. Fetal echocardiography indicated severe pulmonary stenosis with IVS and moderate TR with a jet velocity of 4.7 m/s (Figure 1A). Four weeks later, the woman (at 30 weeks of pregnancy) underwent fetal echocardiography; the results showed a TR jet velocity of 2.56 m/s, pulmonary stenosis, and reversed flow in the ductus arteriosus. The predictors of non-BV outcome related to the degree of RV development with no RV sinusoids are shown in Table 1. The fetus had not reached the point of necessitating intrauterine invasive procedures in accordance with the standards put forth by Gómez-Montes et al. in 2011 (4). Fetal echocardiography was repeated every 2–3 weeks and the relevant indicators are detailed in Table 1. All indicators can be summarized by the following characteristics: the severity of RV hypoplasia did not worsen and failed to meet the indicators for an intrauterine intervention; and the degree and velocity of TR decreased significantly.

**Table 1:** Fetal echocardiography data during pregnancy.

Gestational age (weeks)	Pulmonary valve orifice flow velocity (m/s)	Tricuspid regurgitant jet velocity (m/s)	RV/LV	TV/MV	PV/AV	TVI/CC	TV-Z score
26	2.13	4.70	—	—	—	—	—
30	1.90	2.56	0.76	0.88	0.96	0.43	−0.67
32	2.71	1.66	0.78	0.82	0.90	0.39	−0.88
34	3.10	1.58	0.80	0.75	0.92	0.40	−1.30
37	2.70	1.56	0.82	0.72	0.94	0.38	−1.24

—, no data; RV/LV, right ventricle/left ventricle long-axis ratio; TV/MV, tricuspid/mitral annulus ratio; PV/AV, pulmonary valve/aortic valve annulus ratio; TVI/CC, tricuspid valve inflow duration/cardiac cycle ratio.

A female infant was delivered at 38 weeks with a birth weight of 3,100 g, heart rate of 128 beats/min, respiratory rate of 45 breaths/min, and percutaneous arterial oxygen saturation (SPO_2_) of 93%. A bedside echocardiogram was immediately conducted on the infant, which showed the small RV cavity (Figure 1B); the foramen ovale measured 6.4 mm and the pulmonary valve annulus measured 7.6 mm. There was no obvious blood flow through the valve during systole. The patent ductus arteriosus (PDA) measured 4.3 mm and supplied the pulmonary circulation (Figure 1B). The tricuspid valvular annulus was 10 mm with a TV-Z score of −1.13, with a slight amount of blood flow in diastole and mild regurgitation in systole, and no severe abnormalities observed (Figure 1B). The morphology and structure of the aorta (AO) and pulmonary artery (PA) were normal. The female infant was definitely diagnosed as PA/IVS.

On her first day of life, the SPO_2_ of the infant decreased to 85%, and the echocardiography showed that the PDA tended to close. The prostaglandin E1 (1–3 ng kg^−1^ min^−1^) was administered and SPO_2_ was maintained to 92% of the total. The following problems were evaluated repeatedly before the operation: no severe TV malformation (Ebstein's anomaly); the TR jet velocity was maintained at 2.0 m/s; no myocardial sinusoids; and the coronary artery was normal. Radiofrequency-assisted perforation is a feasible valvotomy option in catheterization laboratories with proper equipment; cost limitations and a considerable success rate are essential in developing countries, explaining the decision to opt for balloon catheter dilation. In addition, the possibility of ductus-dependent pulmonary blood supply is low after percutaneous balloon pulmonary valvuloplasty.

### 2.2 Cardiac catheterization

On the infant’s 20th day of life, she underwent a catheterization intervention. The right ventriculography confirmed pulmonary atresia and the presence of three parts of RV (the inflow, outflow, and trabecula) (Figure 1C). The aortogram showed that the ductus arteriosus was measured approximately 4.5 mm, and the origin and course of the coronary artery were normal. Cardiac catheterization manometry has shown that the RV was 67/10(14) mmHg, AO was 59/25(39) mmHg and PA was 35/22(28) mmHg.

A 4F goose neck snare was manipulated from AO through PDA and kept opening against the pulmonary valve. The soft end of the available coronary wire (Conquest Pro 8–20 wire) was gently manipulated and advanced along the catheter, keeping both the wire and the catheter close to the membranous valve. The wire was required to be pushed very gently to perforate the valve. Once the wire perforated the membrane, the guidewire was manipulated into the PA, the snare grabbed the guidewire, further advancing carefully through the PDA into the descending aorta, and was then exteriorized through the femoral arterial sheath, creating an arteriovenous wire loop and achieving stable position (Figure 1C). A coronary balloon measuring 1.25 mm × 15 mm was maneuvered over the wire to perform the first dilatation. The balloon size was gradually increased from 2.5 mm to the maximum of 8 mm (Figure 1C). At the end of the procedure, the results of the cardiac catheter manometry were as follows: RV 53/13(32) mmHg; AO 52/21(32) mmHg; and PA 34/20(27) mmHg.

After the intervention, the infant was given a continuous dose of prostaglandin E1, and her SPO_2_ was 92%. The oxygen saturation was significantly reduced when the dose of prostaglandin E1 was downregulated. Multiple echocardiography showed no antegrade blood flow across the pulmonary valve (Figure 1D), which was considered to be RV insufficiency, and the pulmonary circulation was dependent on the PDA. After 7 days of intervention, the infant’s SPO_2_ level dropped drastically to below 60%, and an echocardiography indicated that the PDA was closed; an increased dose of prostaglandin E1 was not effective.

A Blalock–Taussig shunt or Glenn's surgery was proposed as the default course of action in emergency scenarios, while the ultimate surgical technique was based on the RV function. The families were unwilling to accept the potential negative long-term outlook and the failure of BV circulation and chose to end the treatment with regret.

## 3 Discussion

The pathophysiological characteristics of severe pulmonary valve stenosis with a tendency toward atresia are similar to those of PA/IVS. We presented a fetal case with severe pulmonary valve stenosis and RV of moderate dysplasia in size and morphology. As the pregnancy progressed, there was a considerable drop in the velocity of fetal TR. After birth, the morphology and size of RV and TV were examined to assess mild to moderate RV dysplasia, although TR velocity was at a low level. There were no myocardial sinusoids and severe TV malformation after repeated evaluations. However, cardiac catheterizations indicated the low RV pressure before and after decompression (approximately 60 mmHg), and RV function was severely deteriorating. The ejection functions could not be supported by the RV, and the flow of blood in the pulmonary circulatory system was dependent on PDA. This may be the first report of a case of fetal severe pulmonary valve stenosis with RV dysfunction when the RV is appropriately dysplastic.

Anomalous RV morphology in cases of PA/IVS is often irregular and singular, which raises the debate on how to assess the severity of RV development. The most effective compromise between sensitivity and specificity predicting a non-BV management pathway are TV/mitral valve ratio ≤0.83, pulmonary valve/aortic valve ratio ≤0.75, tricuspid inflow duration/cardiac cycle length ≤36.5%, and RV/left ventricle length ratio ≤0.64. If three out of four markers are present, this predicts a non-BV outcome with a sensitivity of 100% and specificity of 92% (it is 100% if the four requirements are met) (4). Unfortunately, there are cases where fetal RV decompression is performed, but the patients fail to achieve BV circulation, and an even greater proportion of cases where multiple reoperations are necessary after RV decompression.

Obstruction of the antegrade flow of the RV of PA/IVS causes a high-pressure state in the RV cavity, which is often accompanied by severe TR (5). With the amount of blood entering the RV cavity before decompression being equal to the TV regurgitation volume, TR may aid in guiding prenatal counseling regarding postnatal outcome in PA/IVS (4). In addition, as TR is easily identified by fetal color Doppler during routine obstetric sonography, these patients might come to notice the practitioner more often. Yu et al. diagnosed PA by an expected finding in severe TR in the first trimester prenatal screening (6). Fetal studies have implied that the outcomes after decompression of RV in infants with PA/IVS are associated with the degree of the TR (7). The absence or presence of mild baseline TR is a contributing factor to not reaching the desired BV circulation outcome (8). Lacobelli et al. demonstrated that the absence of TR in fetal PA/IVS correlated to single ventricle circulation postnatally in addition to ventriculocoronary connections (9). Abnormal coronary circulation was associated with severity of TV and RV dysplasia in both fetal and neonatal patients with PA/IVS. In cases of small RV cavity and severe dysplasia, a higher pressure increases the probability of RV-to-coronary flow with fistulous connections. Once the fistulous connections are formed, the pressure in the RV cavity decreases and TR is naturally reduced. Conversely, the absence of TR is highly predictive of fistulous connections in fetuses affected by PA/IVS and is even more reliable at predicting such connections than direct visualization (10). However, in this case, although TR decreased, the formation of a fistula did not occur, and the structure of the RV was moderate. Therefore, the anatomy of this case is difficult to explain due to the above reasons.

Two prototypes of PA/IVS have been presented: type I, which is more frequent, has pulmonary valvular atresia, dysplasia of the TV with the small annulus, sub-developed RV with hypoplastic cavity and hypertrophy, atrial septal defect, and PDA; type II, which is less common, is associated with more severe dysplasia of the TV and enlargement of the RV, right atrium (RA), and right atrioventricular junction, and a notable thinning of the RV wall (11). Choi et al. described a case of fetal PA/IVS with moderate RV dysplasia and enlarged RA. The heart exhibited a dysplastic thickened TV with the leaflet directly attached to the papillary muscle without chords. This is different from types I and II, which is classified as a separate type (type III) (12). Because the specimen is a fetal heart, the RV may not be given adequate time to develop a hypertrophic wall, necessitating further sample collection to ascertain the type III hypothesis.

## 4 Conclusion

PA/IVS is a heterogeneous disease with various degrees of RV dysplasia. Mild or no baseline TR is a reliable indicator with non-BV outcomes for fetuses with PA/IVS, even with the acceptable dysplasia RV structures.

## Data Availability

The original contributions presented in the study are included in the article/Supplementary Material, further inquiries can be directed to the corresponding author.
